# Evaluation of Changes in the Levels of Firmicutes and Bacteroidetes Phyla of Sheep Feces Depending on the Breed

**DOI:** 10.3390/ani10101901

**Published:** 2020-10-16

**Authors:** Paulina Cholewińska, Magdalena Wołoszyńska, Marta Michalak, Katarzyna Czyż, Witold Rant, Marzena Janczak

**Affiliations:** 1 Institute of Animal Breeding, Wroclaw University of Environmental and Life Sciences, 51-630 Wroclaw, Poland; katarzyna.czyz@upwr.edu.pl (K.C.); marzena.janczak@upwr.edu.pl (M.J.); 2 Department of Genetics, Wroclaw University of Environmental and Life Sciences, 51-630 Wroclaw, Poland; magdalena.woloszynska@gmail.com; 3 Department of Animal Nutrition and Feed Management, Wroclaw University of Environmental and Life Sciences, 51-630 Wroclaw, Poland; marta.michalak@upwr.edu.pl; 4 Institute of Animal Breeding, Warsaw University of Life Sciences—SGGW, 02-786 Warsaw, Poland; witold_rant@sggw.edu.pl

**Keywords:** ruminants, sheep, breed, microbiome

## Abstract

**Simple Summary:**

The microbiome plays an important role in the digestive system of ruminants. It affects the health status of animals and their development and production rates. However, its composition may be influenced by factors such as diet, age, gender, and health condition. The study was conducted on three breeds of sheep that were kept in one environment and fed with the same feed. The microbiological analysis showed that the animal microbiome is also influenced by breed.

**Abstract:**

Studies carried out so far have indicated the effect of the microbiome on the composition of ruminant products. Recent studies have shown that not only diet, but also genetic factors can affect the microbiological composition of the digestive system. The aim of the study was to determine the differences in the levels of selected bacterial phyla in terms of breed differences. Three sheep breeds, i.e., Olkuska, Romanov, and old-type Polish Merino, differing in their use (meat–wool, meat, prolificacy) and country of breed origin were included in the study. Sheep at the same age and of the same sex were kept for a period of 3 months in the same environmental conditions and fed the same feed in the same proportions. The study included real-time PCR (polymerase chain reaction) analysis of feces collected before the slaughter and measurements of body weight and chilled carcasses. The obtained results showed significant differences between the breeds in the levels of bacterial populations tested. There were also differences in body weight between the breeds during the first weight measurements, however, the final results did not show any differences—after three months of maintenance all of them reached similar body weights, despite differences in fecal microbiological composition. The study suggests that in addition to diet and environmental conditions, the microbiology can also be influenced by breed.

## 1. Introduction

There are about 200 ruminant species worldwide. The term ruminant is related to the method of plant digestion and forestomach development [1]. This group of animals is also characterized by having a very extensive and diverse microbiome of the digestive system. These microorganisms enable ruminants to absorb nutrients from the most complex polysaccharides, i.e., cellulose and hemicellulose, because the animals themselves do not have the appropriate enzymes in their digestive system [1,2,3]. Through degradation and fermentation, these microorganisms decompose plant parts mainly into volatile fatty acids (VFAs) and other nutrients, providing 70% of their daily energy requirement. In addition, studies conducted so far have shown a significant impact of the microbiological composition of the digestive system on the health status of the animals and the quality of the products obtained from them, i.e., milk and meat, as well as the amount of methane produced [3,4,5]. Anaerobic and relatively aerobic bacteria (mainly Firmicutes and Bacteroidetes) are the most abundant, along with much smaller quantities of Proteobacteria, Fibrobacteres, Tenericutes, and Actinobacteria, followed by archeons (mainly *Methanobacterium*, *Methanosarcina*, and *Methanobrevibacter* species), protozoa (i.e., genus *Isotrichia*, *Dasytrichia*, *Entodidium*, *Diplodinium*, *Endidiplodinium*) and fungi (e.g., *Anaeromyces*, *Caecomyces*, and *Cyllamyces* [6].

According to the literature, the microbiological composition is mainly influenced by factors such as the environment, age, physiological state, and nutrition, and even geographical differences [1,6]. However, ruminants (cattle, sheep, goats) differ not only in terms of species but also breed, and this may be one of the factors differentiating the microbiological compositions of their digestive systems. Not much is known about the influence of breed or even genotype on the microbiological composition of the gastrointestinal tract [6]. Recent studies have shown the influence of the host itself on microbial composition. For example, it was demonstrated that animals differ in the rumen microbiome according to gender and temperament, which may suggest that the genetics and physiology of the host are correlated with the microbiological structure of the digestive system [7,8]. Additionally, the influence of the sire breed in the case of meat cattle on the gastrointestinal microbiome composition of the offspring was demonstrated [9].

However, the differences in terms of breed are not fully understood. There are many species of ruminants in the world, and each of them includes numerous breeds [1]. For example, there are about 14 breeds of sheep in Poland, including Olkuska, Romanov, and old-type Polish Merino sheep [10]. The mentioned examples of breeds differ from each other, for example in their use and maintenance preferences. One of the breeds used in the study was a Polish Olkuska sheep, which is a prolificacy–meat breed that was created in the Olkusz district (Poland) in the 19th century [11,12]. The sheep have a structure typical for dairy animals, they have a large head and ears, they are polled, and they have moderate musculature. Their wool is uniform and white, with loose staples. Olkuska sheep are characterized by their high prolificacy (about 200%) and well-developed maternal instinct. Rams and ewes reach sexual maturity at the age of 10 months. Polish Olkuska is a seasonal breed. In 2005, it was included in the Genetic Resources Protection Program because of the gene responsible for their high prolificacy and good adaptation to the environment in Poland. The aim of this program is to stabilize and preserve the unique breed genotype, increase the population, and maintain genetic variability [10,11,12].

Romanov sheep are imported from Russia. This breed comes from short-tailed sheep living in northern Russia. These sheep are of medium size and have mixed wool, whereby the field of growth does not cover head, legs, or belly. They have a dark-colored coat, while the head and limbs are black with white spots. This is an early maturing breed with very high prolificacy (250%) and asesonality. Currently, this breed is maintained because of its prolificacy, and in past the skin was used to produce coats [10].

The old-type Polish Merino is a sheep with a strong body constitution. The wool has a merino character, whereby the growth area does not include the face and lower limbs. It has well marked meat features and a strong herd instinct. It is an early maturing, asesonal breed with prolificacy at the level of 125%. As with the Olkuska sheep, in 2008 it was included in the Genetic Resources Protection Program due to its low number and very good adaptation to local conditions. For this breed, the objectives of the program are to maintain the population at the level of 9000, to maintain genetic variability and stability, to create a separate herd book, to promote breeding under conditions of sustainable agriculture, and to promote products derived from this breed [10,12].

Therefore, this study focuses on three breeds of sheep kept in the same environment, fed with feed of the same composition, and of similar age, in order to eliminate factors related to diet, environment, health, age, and physiological conditions (before entering the reproductive period). A real-time PCR (polymerase chain reaction) analysis was performed for the most numerous bacterial phyla—Firmicutes and Bacteroidetes—taking into account the share of the Lactobacillaceae family (phylum of Firmicutes).

## 2. Materials and Methods

### 2.1. Material

The animal material consisted of sheep of three breeds: Olkuska sheep (O), old-type Polish Merino sheep (M), and Romanov sheep (R). All sheep were kept for a period of 3 months at the Rams Evaluation Station (RES) in Kociugi (GPS: 51.834507, 16.788538; Poland), an affiliate of the National Research Institute of Animal Production in Pawłowice, Poland (GPS: 51.823696, 16.752217; Poland).

The sheep were brought to the station at the age of 3 months (all kept in Poland). They were placed in group boxes of 10 heads per breed and maintained in an indoor system. All animals were fed with ready-made grain pellets (about 300 g/head/day) and had access to hay ad libitum. The composition of the ready-made grain pellets and the daily dose were determined in accordance with the standards of ruminants nutrition [13]. The animals were of one sex (rams) and were characterized by having good health status (without the symptoms of disease, e.g., diarrhea, fever). They were kept at the station (all in the same building and conditions: temperatures of about 20–25 °C, humidity of 60–70%) for about 3 months until they reached a body weight of 40–42 kg, then were slaughtered for sale. The selected breeds differed both in terms of performance and origin, being both native (Polish) and imported breeds; however, they were all born and maintained in Poland.

### 2.2. Sample Collection

Fecal samples were collected from each individual after 3 months of their stay in the Rams Evaluation Station. They were collected in sterile containers up to 10 s after defecation (100 mL), transported to the laboratory in a thermal container (at –5 °C), then frozen to −26 °C until analysis (about 2 months).

### 2.3. Evaluation of Ram Body Gains

On arrival at the station and at the end of the study, the rams were weighed in order to determine body weight gains and daily gains. Differences in body weight were also evaluated before the slaughter and chilled carcasses were assessed in terms of breed differences.

### 2.4. Isolation of DNA

The Genomic Mini AX Stool kit (A&A Biotechnology, Gdańsk, Poland) was used for DNA isolation, modified by the addition of mutanolyzes and lysozyme. Then, after isolation, the quality of the obtained DNA was checked with the NanoDrop 2000 spectrophotometer from Thermo Scientific (Wilmington, NC, USA). The average DNA content was 80–100µg/µL. The level of impurities in the samples was at the level for parameter 260/230:2.0–2.2 and for parameter 260/280:1.8–2.0. In the case of high levels of contamination or possibly low-quality DNA, the samples were re-isolated or cleaned with the Clean-up Concentrator (A&A Biotechnology).

### 2.5. Real-Time PCR Analysis

Real-time PCR analysis was performed with the use of a Bio-Rad CFX Connect 96 Touch apparatus with the help of the SsoAdvanced™ Universal SYBR^®^ Green Supermix kit (Bio-Rad Laboratories, Inc., CA, USA) at a volume of 10 μL in 3 technical repetitions (Table 1). A no template control (NTC; without DNA sample, only primers) test was additionally performed for each gene. The real-time PCR analysis strategy was based on the amplification of genes specific for the tested phyla against the reference gene for all bacteria. The reference genes were 16S universal eubacterial genes 530F (5′-GTC CCA GCM GCN GCG G) and 1100R (5′-GGG TTN CGN TCG TTG) [14], for the Firmicutes phylum these were 16S 928F-Firm (5′-TGA AAC TYA AAG GAA TTG ACG) and 1040FirmR (5′-ACC ATG CAC CAC CTG TC), and for Bacteroidetes these were 16S 798cfbF (5′-CRA ACA GGA TTA GAT ACC CT) and cfb967R (5′-GGT AAG GGT TCC TCG CGT AT) [15]. For the *Lactobacillaceae* family the used genes were 16S, lac1 forward (5′-AGC AGT AGG GAA TCT TCC A), and Lac2Seq (5′-ATTTCACCGCTACACATG) [16].

A standard curve was prepared for the genes tested to determine the performance of individual genes. A sample dilution of 10^−6^ from the 10^−2^ to 10^−7^ series of dilutions was selected for analysis. The analysis was performed according to a protocol of 40 cycles: polymerase activation and DNA denaturation 95 °C (3 min), denaturation 95 °C (15 s), annealing 60.5 °C (15 s), extension and plate reading at 72 °C (40 s). The analysis of the melting curves for the samples was performed at temperatures ranging from 65 °C (5 s) to 95 °C (0.5 °C increments in 2 s). The data were then compiled using the CFX Maestro software (Bio-Rad Laboratories, Inc., CA, USA). The sample with a DNA level of 100 μg/μL and impurities at a level in line with the above mentioned standards was an arbitrary calibrator. CFX Maestro calculated the results from the number of reference gene matrixes and the differences at the relative normalized expression (ΔΔCq–RNE) phylum level, taking into account the amplification efficiency levels of individual genes.

### 2.6. Statistical Analysis of the Results

The obtained results were analyzed using Statistica ver. 13.1 (Statsoft, Krakow, Poland). The data distribution was verified using a Shaphiro–Wilk test. As the distribution was normal, the one-way ANOVA test (breed) was used. Differences in the ANOVA test were determined using the Tukey test (*p* > 0.05). Each test was performed in duplicate.

## 3. Results

Body weight measurements made after transporting the animals to the Rams Evaluation Station (RES) showed significant differences between the Olkuska sheep (O) and old-type Polish Merino (M) breeds and Romanov sheep (R). The Olkuska sheep breed was characterized by having the highest average body weight of about 20.59 kg, followed by the old-type Polish Merino (18.43 kg on average), while the lowest weight was found in Romanov sheep at 7.39 kg on average (Table 2). No significant breed differences were found in the case of weight measurements before the slaughter (after a period of 3 months) and in the carcass weight after chilling. Significant differences were shown in the case of daily gains, where Romanov sheep were characterized by higher daily gains compared to Olkuska sheep.

**Figure 1:**
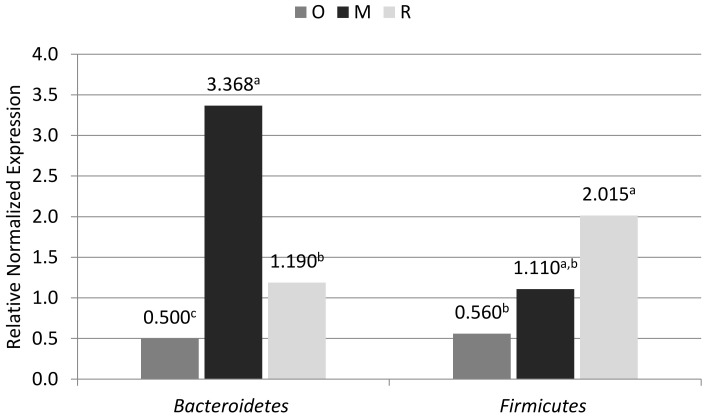
Levels of Bacteroidetes and Firmicutes in feces from Olkuska sheep (O), old-type Polish Merino sheep (M), and Romanov sheep (R). *p* < 0.05—a, b, c (for Bacteroidetes *p* = 0.0037/0.0053; for Firmicutes *p* = 0.019).

In the case of real-time PCR analysis, significant differences between the breeds were found in the phylum levels (relative normalized expression—RNE). The greatest differences were noted in the RNE levels of Bacteroidetes. The highest level was found in the old-type Polish Merino, while the lowest level was found in the Olkuska sheep. In the case of Firmicutes group, there were significant differences between the Olkuska (the lowest level) and Romanov sheep (the highest level). In the case of old-type Polish Merino sheep, the level of Firmicutes group was in between the other two breeds (Figure 1).

**Figure 2:**
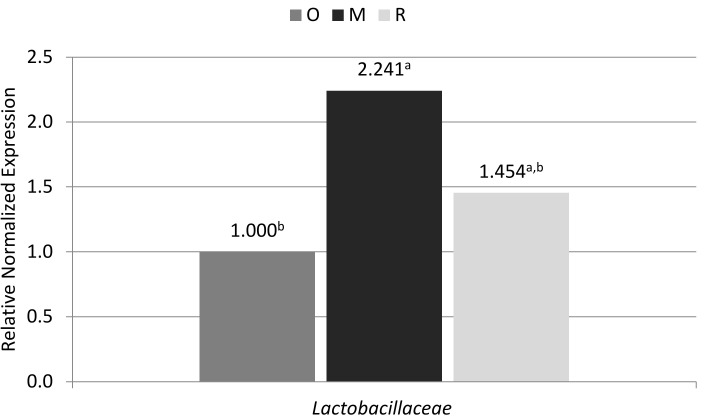
Level of Lactobacillaceae in feces from Olkuska sheep (O), old-type Polish Merino sheep (M), and Romanov sheep (R). *p* = 0.0102.

Analyzing the results of real-time PCR for the Lactobacillaceae family (Firmicutes phylum), the inverse relationship was shown in the examined individuals as for the whole Firmicutes group. Olkuska sheep were characterized by have the highest level of RNE for Lactobacillaceae, while the lowest level was found for Romanov sheep. However, as in the previous case, the level of bacteria in the old-type Polish Merino was intermediate (Figure 2).

In terms of the proportions of Bacteroidetes and Firmicutes phyla, the highest value was demonstrated in the case of old-type Polish Merino at about 3.5:1, while the smallest differences between the phyla were noted in the Olkuska sheep (1:1). In the case of Romanov sheep, the ratio was 0.6:1, which indicates a lower level of Bacteroidetes with respect to Firmicutes.

## 4. Discussion

A recent study by Hernandez-Sanabria et al. [9] showed that not only do the paternal genes have an effect on microbial composition in ruminants, but also the breed. Although ruminant species generally have similar levels of major bacterial phyla, there are inter-breed differences. A study on probiotics conducted in the 1980s showed differences in the bacterial compositions of goats of different breeds and living environments in terms of *Synergistes jonesi*. The bacterium was found in goats in Hawaii, but was not detected in goats from Australia. Goats with this bacterium could digest myosin in the *Leucaena* shrub, which was also found in Australia. The authors of the study conducted an experiment in which the bacterium was introduced into the digestive system of Australian goats, which later enabled them to digest myosin [17].

As can be concluded based on this study, one of factors that can affect the microbiology of the digestive system is the breed of the animal; the breed is related to the selection in order to adapt animals to their living conditions. In this study, one of the breeds, namely Romanov sheep, was bred in Russia and imported to Poland. As can be noted, despite the already long period of maintenance in Poland, its microbiological composition differed in comparison to the breeds kept and selected in Poland, especially in relation to the Olkuska sheep. The Romanov sheep differed in the levels of the Bacteroidetes phylum compared with the old-type Polish Merino and in the levels of Firmicutes phylum compared with the Olkuska sheep. In the feces of Romanov sheep, the level of Bacterioidetes was lower than in the old-type Polish Merino, but was also slightly higher than in the Olkuska sheep, while in the case of Firmicutes the level was the highest compared to other examined breeds (where it was significant in relation to the Olkuska sheep). The Romanov breed also differed from the Olkuska breed in terms of the level of the Lactobacillaceae family (a phylum of Firmicutes) in the feces. In spite of the higher level of the general Firmicutes in the Romanov breed, the Olkuska breed was characterized by a significantly higher level of the Lactobacillaceae family. The obtained results may suggest that although the Romanov sheep as a breed was in the past formed and selected for desirable traits in Russia and was then subject to conditions prevailing in this country, this could have affected the composition of its current microbiome. Despite being maintained in Poland, this could still contain the features of the microbiome from the time of breed formation in Russia [6,18,19,20].

In the case of the Olkuska breed, on the other hand, the ratio of selected bacterial phyla (Firmicutes-to-Bacteroidetes) was 1:1, while in the Romanov sheep it was 0.6:1. The results obtained could be related to the diet used in the case of sheep kept for meat and milk. They are often kept in large flocks, and the Olkuska and Romanov sheep are poorly adapted to such a system of maintenance [21,22]. Additionally, in large flocks, often in conditions of intensive breeding, animals receive more concentrated feed in the diet than in extensive breeding based mainly on roughage [11,21]. In the case of animals in which an increase in the amount of concentrated feed is applied, there is an increase in the level of bacteria from the Bacteroidetes phylum and a decrease in the bacteria responsible for the decomposition of roughage, which mainly belong to the Firmicutes phylum [22]. In the case of breeds kept in small flocks, where mainly roughage is applied, the level of phyla responsible for the decomposition of roughage should be higher or similar in relation to the bacteria responsible for the distribution of concentrated feed [4,23,24]. Both the Olkuska and Romanov sheep had similar ratios of the selected bacteria phyla in terms of the examined groups. The obtained results suggest that long-term maintenance of breeds with a diet adjusted to the expected performance resulted in the formation of a microbiome adjusted to a given diet [6,20].

Not many studies have been conducted on breed influence of the on the microbiological composition of the digestive system, and the studies conducted so far suggest differences between the breeds in terms of the microbiological composition of the gastrointestinal tract. The results obtained in this study are likely to be related to the selection effect used to obtain a breed with appropriate production rates for breeders. In the case of the Romanov sheep, this was mainly the prolificacy; in the case of the Olkuska sheep, this was both prolificacy and muscularity; while in the case of the old-type Polish Merino, this was wool quality and muscularity [11,21].

In order to achieve the above-mentioned results, different diets were used in the described breeds and they were kept for many years in different maintenance systems. The studies by Douglas et al. [18] and Xin et al. [24] confirm the theory that there are differences in the microbiological compositions of the digestive system in different ruminant breeds, both in cattle and sheep. It has been suggested that different diets were used in order to achieve the results expected by the breeders and that the animals were maintained for many years in different environments and housing systems. Selection under the influence of factors such as the housing system or diet could influence the adaptation of the animal and its microbiome to the conditions prevailing for many years or even centuries [22,25,26,27].

Interestingly, despite the differences in microbiological composition, the animals achieved similar body weights and chilled carcass weights over a similar time period (about 3 months). This may indicate that despite the difference in microbiological composition between the breeds, the feed was properly absorbed by the animals. The obtained results suggest that the diet, despite the general influence on the composition of the gastrointestinal microbiome, did not change the level of the main gastrointestinal tract phyla in terms of the breed. In addition, in should be emphasized that the results obtained from the feces of ruminants reflect the composition of the majority of the digestive system, because the large intestine is, after the rumen, the most inhabited part of the digestive system by the microbiome [6]. However, further studies on the changes in the microbiome composition in terms of genotype and breed are recommended, taking into account also ruminal microorganisms, as the great majority of fermentation takes place in the rumen, which was certainly the limitation to this study.

## 5. Conclusions

The study demonstrated that despite the same diet and environment, the animals differed microbiologically in terms of breed. However, as the breed differences are not fully understood, further research should be undertaken. Many breeds have been bred for specific purposes (for meet, wool, or prolificacy), but knowledge about the effects of a factor such as the breed may enable the breeders to more accurately match animals to their environment, diet, and selected performance indices for best results.

## Figures and Tables

**Table 1:** Proportion of PCR mix.

Component	Volume per 10 μL Reaction
SsoAdvanced™ Universal SYBR^®^ Green Supermix	5 μL
Forward and reverse primers	1 μL (0.8 μM)
DNA template	2 μL (0.04–0.015 ×10^−4^)
Nuclease-free water	2 μL

**Table 2:** Weight of rams and carcasses after cooling.

Time	Romanov Sheep		Old-Type Polish Merino Sheep		Olkuska Sheep		*p*-Value
	Average	SEM *	Average	SEM	Average	SEM	
On the day of arrival	17.39 ^A^	0.7	18.43 ^a^	0.27	20.59 ^B, b^	0.67	0.001/0.038
Before slaughter	40.12	0.30	40.03	0.37	40.80	0.56	0.51
Carcass after cooling	18.69	0.35	18.67	0.34	19.73	0.37	0.09
Daily growth	0.25 ^a^	0.009	0.24	0.005	0.23 ^b^	0.004	0.017

Note: *p* > 0.05—a,b; *p* > 0.01—A,B; * Standard Error of the Mean.
